# The European Nucleotide Archive in 2018

**DOI:** 10.1093/nar/gky1078

**Published:** 2018-11-05

**Authors:** Peter W Harrison, Blaise Alako, Clara Amid, Ana Cerdeño-Tárraga, Iain Cleland, Sam Holt, Abdulrahman Hussein, Suran Jayathilaka, Simon Kay, Thomas Keane, Rasko Leinonen, Xin Liu, Josué Martínez-Villacorta, Annalisa Milano, Nima Pakseresht, Jeena Rajan, Kethi Reddy, Edward Richards, Marc Rosello, Nicole Silvester, Dmitriy Smirnov, Ana-Luisa Toribio, Senthilnathan Vijayaraja, Guy Cochrane

**Affiliations:** European Molecular Biology Laboratory, European Bioinformatics Institute, Wellcome Genome Campus, Hinxton, Cambridge CB10 1SD, UK

## Abstract

The European Nucleotide Archive (ENA; https://www.ebi.ac.uk/ena), provided from EMBL-EBI, has for more than three decades been responsible for archiving the world's public sequencing data and presenting this important resource to the scientific community to support and accelerate the global research effort. Here, we outline ENA services and content in 2018 and provide an overview of a selection of focus areas of development work: extending data coordination services around ENA, sequence submissions through template expansion, early pre-submission validation tools and our move towards a new browser and retrieval infrastructure.

## INTRODUCTION

For over a third of a century, the European Nucleotide Archive (ENA) has served as a cornerstone of the world's bioinformatics infrastructure. In 2018, the resource continues as a broad and heavily used platform for the sharing, publication, safeguarding and reuse of globally comprehensive public nucleotide sequence data and associated information.

ENA content spans a spectrum of data types from raw reads to asserted annotation and offers a diverse portfolio of services to the scientific community. Submissions services cater for our broad range of data providers, from the small-scale submitting research laboratory to the major sequencing centre. Data access services, such as our search and retrieval interfaces, support users from those browsing casually to those integrating ENA content through programmatic access into their own analyses and software applications. Our Helpdesk provides support to several thousand active data submitters and many times this number of data consumers.

As a member of the International Nucleotide Sequence Database Collaboration (1) (INSDC; http://www.insdc.org/), we partner with the National Institute of Genetics’ DNA DataBank of Japan (2) and the United States National Center for Biotechnology's GenBank and Sequence Read Archive (3). Together we provide globally comprehensive coverage through routine data exchange, to build the scientific standards for this exchange and to promote the timely sharing of well-structured sequence data.

Throughout 2018, we have continued to provide ENA services for the submission, archiving, discovery and retrieval of globally comprehensive data across sequencing platforms and scientific applications. In addition to operation and support of ENA, a key focus for the year has been the extension of ENA core service provision (see Table 1) and involvement in data coordination activities around the services.

**Table 1.** Suite of ENA services

Service class	Service	Purpose of service
Data submission	Submissions entry point	Interactive link to various submission tools
User support	Support entry point	Link to Help desk, training and documentation
Data access	ENA Browser	Web-based and RESTful programmatic data retrieval
	Advanced Search	Search interface
	Sequence Similarity Search	Search interface
	Discovery API	Search interface
	ENA File Downloader	High-volume data access service

## SUMMARY OF ENA CONTENT, SERVICES AND COMMUNITY SUPPORT

This year we have continued to provide through ENA a rich set of open services and responsive support for the submission, archiving, enrichment and presentation of the world's sequencing data across an ever increasing range of data types, scientific applications and user communities. Under the Webin submission services framework, we continue to operate both interactive and programmatic interfaces for the submission of data of all accepted types. In the year we have processed 6600 studies, from some 800 000 samples, comprising 600 000 libraries, 31 000 assemblies and 2000 sets of shorter sequences that are not parts of any assemby. We continue to support several thousand active data submitters through our dedicated helpdesk support system that handles 5000 helpdesk tickets annually. We provide extensive user documentation and tutorials (https://ena-docs.readthedocs.io/en/latest/index.html) and offer a number of in-person and online training courses (https://www.ebi.ac.uk/ena/support). We provide secure and permanent archiving for submitted data and for data contributed by our INSDC partners, through EMBL-EBIs technical infrastructure of multi-site secure and modern data centres. To date we have accumulated a globally comprehensive set of 1.5 × 10^9^ sequences and 8 × 10^15^ base pairs of read data across 1.5 × 10^6^ taxa.

Data are presented through a suite of services listed in Table 1. The centrality of ENA to bioinformatics infrastructure has been recognized in our certification as an ELIXIR Core Data Resource (4) (https://www.elixir-europe.org/platforms/data/core-data-resources) and ELIXIR Deposition Database (https://www.elixir-europe.org/platforms/data/elixir-deposition-databases).

## DEVELOPMENT OF DATA COORDINATION SERVICES AROUND ENA

We have continued to operate and grow our portfolio of data coordination services, in which we leverage ENA’s technical platform to support partners in data sharing, analysis, archiving and publication across a breadth of scientific areas. While data coordination is an important bioinformatics service in its own right with clear value for its users, it is also key to the core ENA operation as it serves to extend and improve ENA content and stimulate community-engaged data standards development.

We focus on collaborative projects in which we partner with members of scientific communities to coordinate high-quality standardized content and manage cloud-enabled data processing and analysis through such tasks as the provision of checklists, data validation tools and analysis workflow environments. During the year, for example, we have developed sample checklists targeting food-borne and clinical bacterial pathogen surveillance, tools for the validation and reporting of data from anti-microbial resistance profiling and, with our partners in the COMPARE project, workflows for core genome MLST analysis on these isolates (http://www.compare-europe.eu/). Our broad portfolio of data coordination projects in 2018 covered infectious disease, agriculture, metagenomics, biodiversity, marine science and stem cell biology (see https://www.ebi.ac.uk/ena/about/projects-and-collaborations for further information on these projects). We welcome contact from those working across the life sciences who wish to collaborate with us in areas where our data coordination services might be useful. A future focus in this area will be the development of a data partitioning system that will allow rule- and list-based definitions of ‘slices’ of ENA content relevant for a given analysis application and the presentation of the defined partition of data for user download or cloud compute access. The data partitioning system is at this point in early design; as we finalize the functions and services to be provided from our data partitioning system, we plan to make announcements through our web, e-mail and Twitter channels as to future functionality.

## PRE-SUBMISSION VALIDATION AND THE COMMAND-LINE INTERFACE

The traditional model for data submission to ENA is for submitters to prepare their data, with reference to our documentation, and submit these prepared data through one of our submission interfaces. Under this model, most data validation is applied post-submission, and the feeding back of errors and subsequent coordination of corrected submissions occupies a significant fraction of Helpdesk time. This can lead to delays and frustration for submitters, and increased workload for ENA Helpdesk staff.

Offline pre-submission validation promises a far more direct accessibility to submitters of validation results and hence a more rapid and independent cycle of correction, revalidation and submission. Indeed, it has been a successful strategy for other EMBL-EBI resources, such as PRIDE (5). We are therefore developing tools to support pre-submission validation in order to enhance the submitter experience and reduce Helpdesk overhead. Pre-submission validation is a key principle for EMBL-EBI’s emerging cross-archive unified submissions interface, and in addition to immediate submitter utility, our tools will ultimately provide technical components into this broader platform. Data brokers, such as Genoscope (http://jacob.cea.fr/drf/ifrancoisjacob/Pages/Departements/Genoscope.aspx) and GFBio (https://www.gfbio.org/), will also benefit from these tools in the support they provide to their users. Over the year, we have developed a command-line pre-submission validation tool (https://github.com/enasequence/webin-cli) for the major ENA submission types (genome assemblies, sequencing reads and transcriptome assemblies). Validation checks can be categorised into (i) file format, (ii) file semantics, (iii) inter-file semantics and (iv) assay metadata validation. All categories are amenable to offline validation. In 2018, we identified selected genome assembly submitters for trialling the pre-submission tool and by early 2019 expect to promote the tool broadly to all submitters.

## ANNOTATED SEQUENCE SUBMISSIONS OTHER THAN GENOMES OR TRANSCRIPTOMES

In addition to genome and transcriptome assemblies, Webin supports annotated sequence submissions, such as sets of orthologous loci sequenced across multiple species. We have improved this submission route in Webin to validate much of the annotation before submissions are accepted.This change has reduced time-consuming manual processing of incoming data sets. The new workflow also supports automated post-submission validation and processing of sequence records resulting in faster turnaround and accessioning times. All sequence annotation checklists are supported from the previous system (http://www.ebi.ac.uk/ena/submit/annotation-checklists). These checklists are tables, which ones filled, are transformed into flat file records. The checklists collect the minimum information required for constructing the flat files as well as additional fields selected by the submitters.

**Figure 1.**
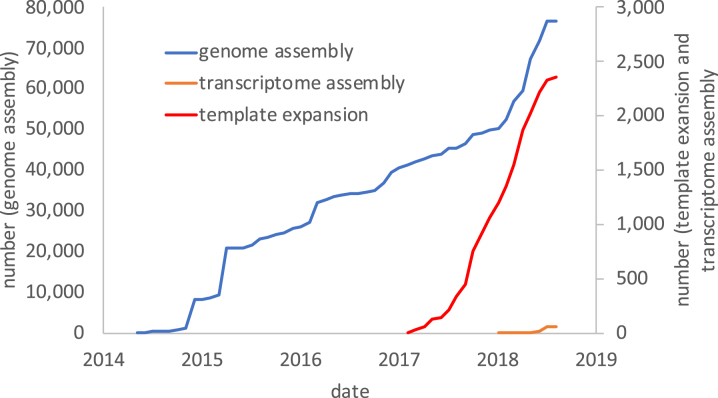
Submissions accumulation, showing template expansion, genome assembly and transcriptome assembly data types.

To date, 2,074 submissions have been completed, representing ∼160 per month, a submission rate for the data type consistent with historical figures since at least 2014. While quality and consistency is retained (through the checklist system), we now achieve the same rate of submission with vastly reduced Helpdesk interventions. Submission rates for template expansion submissions are shown, alongside data for transcriptome and genome assemblies in Figure 1.

## ENA INTEROPERABILITY AND INTERNATIONAL STANDARDS

Active engagement with international standards bodies is essential to further the ENA strategy for interoperability. Major areas of work include our continued engagement with the Global Alliance for Genomics and Health (GA4GH). GA4GH is establishing a common framework of harmonized approaches to enable effective and responsible sharing of genomic and clinical data, not least as one of its accepted ‘driver’ projects with our sister databases, EGA (6) and EVA (7) (https://www.ga4gh.org/howwework/driver-projects.html). ENA also works with the global pathogen genomics community through the Global Microbial Identifier initiative (GMI; http://www.globalmicrobialidentifier.org/) and we continue our close work around microbial diversity and metagenomics with the Genomic Standards Consortium (GSC; http://gensc.org/) (8). We are also continuing to provide data services for the livestock genomics community through the Functional Annotation of Animal Genomes (FAANG; https://www.animalgenome.org/community/FAANG/) initiative (9,10).

## TOWARDS A NEW BROWSER AND RETRIEVAL API: A MODERN PRESENTATION OF THE WORLD’S SEQUENCING DATA

**Figure 2.**
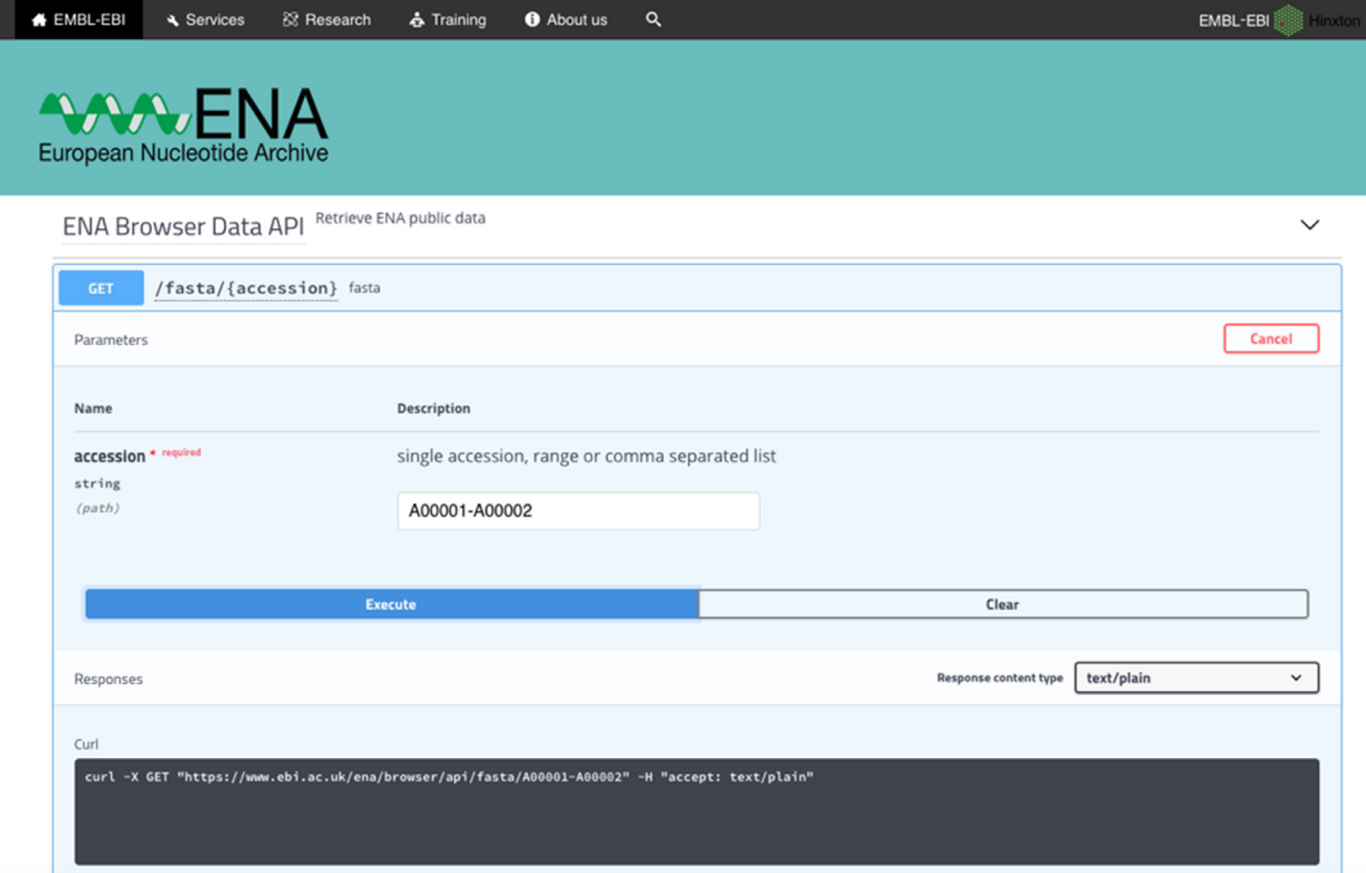
The new ENA Browser Data API swagger interface allowing users to construct and test programmatic interactions.

Throughout 2018, the ENA has continued the development of its modern presentation framework, with the release of its new retrieval API and the upcoming release of a completely redeveloped ENA browser. The new ENA Browser API retrieval service (https://www.ebi.ac.uk/ena/browser/api/) represents a significant improvement in stability and performance over previous offerings. Our previous programmatic service requests for data downloads were handled by the ENA Browser itself and served from a single file system. The data were stored as cumulative files and each request required that the relevant cumulative file be loaded and accessed using location pointers to retrieve each record. Our new service is backed by a MongoDB cluster (https://www.mongodb.com), where each record is stored separately as a document and indexed by accession. Therefore accessing each record by accession is faster and avoids the bottlenecks of the previous system by bypassing the ENA Browser's internal routing. The new storage solution will also support scalability as usage demand increases to continue to provide stable performance. The Browser API no longer has a concept of periodic ‘release’ and instead retains the most recent record of each sequence version. This service will replace the ENA Sequence Version Archive service, by providing available version details for an accession. The API comes with a Swagger interactive interface (https://swagger.io) that allows users to construct and test programmatic queries, as well as access documentation and view available field value terms and restrictions (Figure 2). We now plan to roll out this service to other ENA data types over the coming year.

The development and deployment of our new browser and API, represents a significant change to our traditional data presentation service. Many external services rely heavily on linking and accessing data from the ENA and have developed their own services to interact directly with us over many years. For the scientific resources that rely on retrieval of data from our services, we will ensure appropriate support to these users for the transition to the new browser and retrieval API. For example, our presentation services will no longer support access to files in XML format, so to support our users in the transition period we have developed a tool to allow user conversion to legacy file formats such as XML (https://github.com/enasequence/ena-browser-flatfile-to-xml).

On release of the new browser we will enter an extensive period of user testing, community feedback and iterative improvement. For services that are being replaced or significantly redefined we will maintain a period of overlap where both old and new services are available and will provide extensive documentation and training to support our user bases transition to the new framework. We will continue to announce when new services become available via our news page (https://www.ebi.ac.uk/ena/news), announcements mailing list (http://listserver.ebi.ac.uk/mailman/listinfo/ena-announce) and both the EBI and ENA twitter accounts (@emblebi and @enasequence, respectively). This modernized, fast and simplified framework will provide a stable platform for the continued operation and development of our services to support sequence-enabled science long into the future.

## Data Availability

ENA services are freely available at (http://www.ebi.ac.uk/ena). Content is distributed under the EMBL-EBI Terms of Use available at (https://www.ebi.ac.uk/about/terms-of-use).
